# Effects of α-zirconium phosphate and zirconium organophosphonate on the thermal, mechanical and flame retardant properties of intumescent flame retardant high density polyethylene composites†

**DOI:** 10.1039/d0ra04929h

**Published:** 2020-08-21

**Authors:** Santosh Khanal, Yunhua Lu, Li Dang, Muhammad Ali, Shiai Xu

**Affiliations:** Shanghai Key Laboratory of Advanced Polymeric Materials, School of Materials Science and Engineering, East China University of Science and Technology Shanghai 200237 China saxu@ecust.edu.cn +86-21-64253353; School of Chemical Engineering, Qinghai University Xining 810016 China; Central Department of Chemistry, Tribhuvan University Kirtipur Kathmandu Nepal

## Abstract

The combination of synergistic agents with intumescent flame retardants (IFRs) is an excellent strategy for the development of high-performance flame retardant composites. Zirconium-based compounds are multifunctional materials with applications in various fields. In this study, zirconium-based compounds were synthesized and then combined with an IFR composed of ammonium polyphosphate (APP) and tris (2-hydroxyethyl) isocyanurate (THEIC) to prepare flame retardant high density polyethylene (HDPE) composites. α-Zirconium phosphate (α-ZrP) and two organic–inorganic hybrids (zirconium organophosphonate), Zr-ATMP and Zr-PA, were prepared using amino tri (methylene phosphonic acid) (ATMP) and phytic acid (PA), respectively, and their thermal, mechanical and flame retardant properties were characterized by thermogravimetric analysis, tensile test, limiting oxygen index (LOI) measurement and cone calorimetry test. The results showed that the LOI value of HD/IFR/Zr-ATMP composite reached a maximum of 26.2% using 25 wt% of flame retardant containing 3 wt% of Zr-ATMP. Of the three zirconium-based compounds, Zr-ATMP and α-ZrP can reduce the peak heat release rate compared with the composite containing only IFR. However, zirconium-based compounds showed no significant improvement of tensile strength.

## Abstract 2

Zirconium-based compounds are combined with intumescent flame retardant (IFR) to prepare flame retardant high density polyethylene composites.

## Graphical abstract

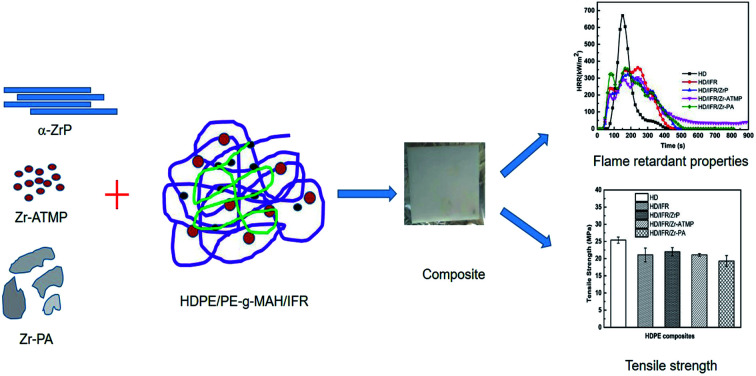

## 1. Introduction

Polymer/nanosheet composites have gained a growing interest in material science due to their unique properties such as high thermal stability, excellent gas barrier properties, and low flammability. Those layered compounds such as montmorillonite (MMT), layered double hydroxides (LDHs), graphene, and layered phosphate are widely used to prepare multifunctional nanocomposites. Particular attention has been given to crystalline zirconium phosphate (α-ZrP) due to its excellent thermal and chemical stability, ion exchange capability, ease of intercalation and/or exfoliation.^1,2^ The formula of α-ZrP is Zr(HPO_4_)_2_·H_2_O and it contains weak Bronsted acid and strong Lewis acid centers derived from the P–OH group and Zr^4+^, respectively.^3^ α-ZrP has many applications in various fields such as catalysis, drug delivery, immobilization of biological materials, proton conductor for fuel cells, anti-corrosion, lubrication, targeted ion removal, and flame retardancy^4,5^.

Polyethylene (PE) is an important commodity plastic with excellent properties such as light weight, excellent electrical insulation, high chemical stability, and ease of processing. However, the highly flammable nature of PE makes it susceptible to fire hazards, and thus there is a need to improve the flame retardant properties of PE-based materials. This can be achieved by adding flame retardant additives, and the intumescent flame retardant (IFR) is regarded as the most promising non-halogenated flame retardant. However, conventional IFRs can be combined with synergistic agents to prepare high-performance flame retardant composites. The use of synergistic nano additives can decrease the loading of flame retardant additives without affecting the mechanical properties.^6–9^

Recent research has suggested that α-ZrP is a promising flame retardant additive to improve the flame retardant properties of polystyrene (PS)^10^ and polyvinyl alcohol.^11^ Alongi *et. al.* studied the flame retardancy of PA6, PP, PET, and EVA nanocomposites containing α-ZrP and found that the flame retardant efficiency was strongly associated with the dispersion of fillers, and intercalated nanocomposites usually showed better flame retardancy.^2^ The flame retardancy of polymer nanocomposites is improved as indicated by the low peak heat release rate (PHRR) in cone calorimeter test (CCT), but they usually fail to pass small scale laboratory tests like limiting oxygen index (LOI) and UL-94 rating. A combination of classical flame retardant additives and α-ZrP as a synergistic agent is a promising way to endow the composites with excellent flame retardant properties. A study was conducted to investigate the effects of melamine and melamine phosphate modified α-ZrP and the IFR system composed of melamine pyrophosphate (MPP) and pentaerythritol (PER) on the flame retardant properties of PP composites.^12^ In another study, the melamine cyanurate complex (MCA) modified α-ZrP was used as a synergistic agent to prepare flame retardant PP composites containing MPP and PER.^13^ α-ZrP as a solid acid can catalyze the dehydrogenation of the polymer and improve the crosslinking and graphitization. α-ZrP shows a catalytic carbonization effect in PP composites containing classical APP/PER based IFR system.^14^ To further improve the catalytic carbonization effect of α-ZrP, a macromolecular charring agent decorated with α-ZrP (ZrP-d-MCA) was synthesized and combined with APP to prepare flame retardant PP composites.^15^ Zirconium phosphate also showed a synergistic effect with ammonium hydroxide (ATH) in EVA composites^16^ and LDPE/EVA blend.^17^ During the combustion, ablative reassembling of the phosphate layer may occur on the surface of the polymer matrix, which can act as a physical barrier to heat and oxygen. Thus, α-ZrP can be used as a synergistic flame retardant filler to develop flame retardant composites. To the best of our knowledge, α-ZrP and related compounds have not been used as flame retardants in PE system. However, few reports are available for α-ZrP based PE nanocomposites. For instance, Lino *et. al.* studied the effect of the intercalation of octadecylamine inside the α-ZrP gallery on the properties of HDPE nanocomposites.^18^ In another study, α-ZrP was found to increase the heat distortion temperature of LLDPE by simultaneously enhancing the strength and ductility of nanocomposites.^19^

The effect of inorganic layered compounds on the flame retardant properties of the polymer depends critically on their interaction with the polymer matrix, which can be enhanced by functionalizing the inorganic materials or, more promisingly, using an organic–inorganic hybrid. Zirconium organophosphonates are important inorganic–organic hybrids synthesized by combining the metal framework with organo–phosphonic linkage. These materials have been widely used in catalysis, ion exchange and adsorption due to their excellent properties such as good thermal stability, large surface area, and variability of acidity. Amino tri(methylene phosphonic acid) (ATMP) is a nitrogen containing phosphonic acid, which can be used to prepare organic–inorganic hybrid for use as water tolerable-solid acid catalyst and adsorbent for heavy metal ions.^20,21^ Phytic acid (PA), a natural plant based antioxidant, can also be used to prepare zirconium-based organic–inorganic hybrid for use in intermediate proton conductor.^22^ These organic–inorganic hybrids may have potential applications in flame retardant polymer composites. Recently, an organic derivative of zirconium containing plant-based cardanol was synthesized for use as an additive for flame retardant epoxy composites.^23^ Thus, zirconium-based compounds, zirconium phosphate and organophosphonate, may be potential synergistic flame retardant additives for IFR-HDPE composites.

In this study, we investigate whether zirconium-based compounds could be used as potential flame retardant additives for IFR-HDPE composites. For this, α-ZrP and two organic–inorganic hybrids were synthesized using PA and ATMP as organic moiety and then combined with ammonium polyphosphate (APP) and tris(2-hydroxyethyl)isocyanurate (THEIC) to prepare flame retardant HDPE composites. The thermal and flame retardant properties of the as-prepared composites were characterized by thermogravimetric analysis (TGA), LOI measurement, and CCT. The char residues were characterized by scanning electron microscopy (SEM), energy dispersive X-ray spectroscopy (EDX), Fourier transform infrared spectroscopy (FTIR) and laser Raman spectroscopy (LRS) to elucidate the possible flame retardant mechanism. Volatile gaseous products were analyzed by thermogravimetry-Fourier transform infrared spectrometry (TG-FTIR), and the tensile properties of the composites were also investigated.

## 2. Experimental

### 2.1 Materials

Zirconium oxychloride octahydrate (ZrOCl_2_·8H_2_O) and anhydrous oxalic acid (COOH)_2_ were obtained from Shanghai Titan Chem Co. Ltd, (China). Hydrochloric acid (HCl) and phosphoric acid (H_3_PO_4_) were obtained from Shanghai Lingfeng Chemical Reagent Co. Ltd, (China). PA (70% in water) and ATMP (50% in water) were obtained from Adamas Beta (Shanghai, China). HDPE (density: 0.93 g cm^−3^; melt index: 20 g/10 min @ 190 °C/2.16 kg) and maleic anhydride grafted polyethylene (PE-*g*-MAH) were obtained from Fushun Petrochemical Co., Ltd, (China). APP (phase II, DP > 1000) and THEIC were supplied by Jinan Taixing Fine Chemical Co., Ltd, (China) and Shanghai Macklin Biochemical Co., Ltd, (China), respectively.

### 2.2 Preparation of α-zirconium phosphate (α-ZrP)

α-ZrP was prepared using oxalic acid.^24^ In a typical procedure, 6.58 g (20 mmol) of zirconium oxychloride octahydrate (ZrOCl_2_·8H_2_O) was dissolved in 200 mL of 1 mol L^−1^ oxalic acid solution. The ratio of oxalic acid/Zr was kept at 10 : 1. A given amount of phosphoric acid was added dropwise to the solution (the mole ratio of H_3_PO_4_/Zr was 6). Then, the mixture was kept at 80 °C for 24 h. After that, the products were separated from the solution by centrifuge, washed three times with water and once with ethanol, and then dried at 70 °C for 24 h.

### 2.3 Preparation of zirconium organophosphonates

Two organic zirconium derivatives were prepared using PA and ATMP, respectively. In a typical procedure, 6.58 g (20 mmol) of zirconium oxychloride octahydrate (ZrOCl_2_·8H_2_O) was dissolved in 200 mL of 1 mol L^−1^ oxalic acid solution, and a given amount of PA (the mole ratio of Zr/PA was 1 : 1) or ATMP (the mole ratio of Zr/ATMP was 1 : 2) was added dropwise at 80 °C and refluxed for 24 h. The resulting products were separated from the solution by centrifuge, washed with water and alcohol, and then dried at 70 °C for 24 h. The products are labeled as Zr-PA and Zr-ATMP hybrid in this study.

### 2.4 Preparation of flame retardant composites

HDPE composites were prepared in 50 g batch using the internal mixer (HL-200) at 170 °C and a rotation speed of 60 rpm. The total content of the flame retardants was kept at 25 wt%, which consisted of IFR (APP and THEIC at a weight ratio of 3 : 1) and 3 wt% of zirconium-based compounds. PE-*g*-MAH was used as a compatibilizer at a weight ratio of 2 : 1 to the zirconium-based compounds. A mixture of HDPE and PE-*g*-MAH was blended for 2 min, and then the flame retardants were added and blended for another 6 min. The composites were pressed into sheets using a hot press (BL-6170-A-25J, Baolun Precision Testing Instruments Ltd, Shanghai, China) at 170 °C at a pressure of 10 MPa for 5 min. The compositions of these composites are shown in Table 1.

**Table 1:** Compositions of the flame retardant composites

S. no.	Samples	HDPE (g)	PE-*g*-MAH (g)	IFR^a^ (g)	Zirconium-based compounds (g)	LOI (%)
1	HD	47.0	3.0	—	—	18.1
2	HD/IFR	34.5	3.0	12.5	—	25.2
3	HD/IFR/ZrP	34.5	3.0	11.0	1.5^a^	24.8
4	HD/IFR/Zr-PA	34.5	3.0	11.0	1.5^b^	23.5
5	HD/IFR/Zr-ATMP	34.5	3.0	11.0	1.5^c^	26.2

a IFR = APP and THEIC at a 3 : 1 weight ratio, a = α-ZrP, b = Zr-PA, c = Zr-ATMP.

### 2.5 Measurement and characterizations

The FTIR spectra were recorded on Nicolet 6700 spectrometer (USA). Samples were pressed into pellets after mixing with KBr, and the FTIR spectra were recorded in an optical range from 400 to 4000 cm^−1^ at a scan number of 32 and a resolution of 4 cm^−1^.

The X-ray diffraction (XRD) patterns were obtained using a Rekagu vertical goniometer with CuKα radiation (*λ* = 0.154 nm) generated at 40 mA and 40 kV at a scan rate of 4° min^−1^ and a step size of 0.02°.

TGA was performed using a Netzsch STA 409 PC thermogravimetric analyzer from room temperature to 700 °C at a heating rate of 10 K min^−1^ in a nitrogen and air atmosphere of 40 mL min^−1^, respectively.

Differential scanning calorimetry (DSC) was performed on Q2000 (TA instrument) in a nitrogen atmosphere of 20 mL min^−1^. Test samples (7–9 mg) were heated in an aluminum crucible to 160 °C at a heating rate of 10 K min^−1^, kept at that temperature for 2 min and then cooled to 0 °C. Finally, the non-isothermally crystallized samples were re-heated up to 160 °C at a heating rate of 10 K min^−1^.

SEM-EDX was conducted on a field emission scanning electron microscope (S-4800, Hitachi Japan) connected with an energy dispersive X-ray spectrometer (Quantax 400-30, Tokyo, Japan) at an accelerating voltage of 15 kV. Samples were sputtered with a thin layer of gold before observation.

LRS was conducted using a laser Raman spectrometer (LabRam HR Evolution) with 532 nm helium–neon laser line within 200–3000 cm^−1^ region at room temperature.

The flame retardant properties were determined by LOI measurement and CCT. LOI test was conducted using an oxygen index instrument (JF-3, Jiangning Analysis Instrument Factory, China) with samples of 100 × 6.5 × 3 mm^3^ according to ASTM D-2863. CCT was performed with samples of 100 × 100 × 4 mm^3^ using a cone calorimeter (FTT2000 cone calorimeter) at a heat flux of 35 kW m^−2^ according to ISO 5660-1.

The tensile properties were determined using a universal testing machine (MTS E 44) at a crosshead speed of 10 mm min^−1^ according to Chinese standard GB/T 1040.2 2006. Five samples were examined, and the average values were reported.

TG-FTIR was carried out on a thermogravimeter (Netzsch STA 409 PC) coupled with an infrared spectrometer (Bruker TENSOR). The temperature was raised from 35 °C to 1000 °C at a heating rate of 10 °C min^−1^. The carrier gas was high-purity nitrogen at a flow rate of 70 mL min^−1^ and the temperature of the transfer line was 200 °C. The spectra were recorded per 21 seconds in optical range 500–4000 cm^−1^.

The chemical compositions of Zr and P in Zr-PA and Zr-ATMP were determined by inductively coupled plasma atomic emission spectrometry (Agilent 725 ICP-OES, USA). The emission radiation was measured by photomultiplication at 339.19 and 213.61 nm for P and Zr, respectively. C, H and N elements were analyzed on Vario-EL III elemental analyzer (Germany).

## 3. Results and discussion

### 3.1 Characterization of α-ZrP and zirconium organophosphonates

**Fig. 1:**
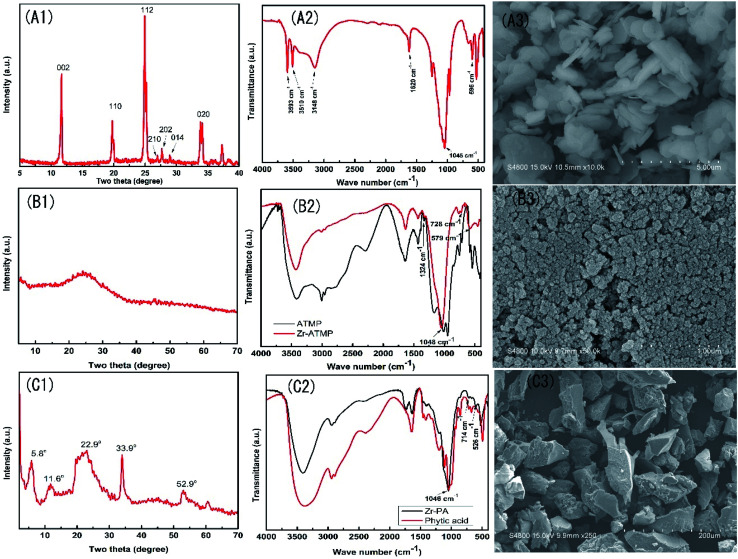
XRD patterns, FTIR spectra and SEM images of α-ZrP (A1–A3), Zr-ATMP (B1–B3) and Zr-PA (C1–C3).

The three zirconium-based compounds were characterized by FTIR and XRD, and the results are shown in Fig. 1. The XRD pattern of α-ZrP (Fig. 1A1) shows a sharp and narrow peak, indicating the crystalline nature of the as-prepared zirconium phosphate. The three characteristic peaks at 11.6°, 19.7°, and 24.9° correspond to the diffraction peaks of (002), (110) and (112) planes, respectively (JCPDS 33-1482). The peak intensity at 2*θ* ≈ 11.6° indicates an interlayer distance of 7.57 Å. In the FTIR spectra of α-ZrP (Fig. 1A2), the peak at 1045 cm^−1^ is assigned to the symmetrical stretching vibration of PO_4_^3−^, the peak at 1250 cm^−1^ is assigned to the P–OH stretching or deformation vibration of HPO_4_^2−^, the peaks at 3593, 3510, 3148 and 1620 cm^−1^ are attributed to the symmetric and asymmetric vibration of water present in α-ZrP, and the peak at 596 cm^−1^ is assigned to the vibration of Zr–O bond^25^. The SEM image (Fig. 1A3) of α-ZrP shows some aggregates of thin platelets.

The XRD pattern of the Zr-ATMP hybrid (Fig. 1B1) shows only a broad peak at around 24°, indicating the amorphous nature of Zr-ATMP. In the FTIR spectra (Fig. 1B2), the strong broad band at 3432 cm^−1^ and the sharp band at 1631 cm^−1^ can be assigned to the hydroxyl groups and surface-adsorbed water, respectively. The bands between 2800–3000 cm^−1^ and the band at 1427 cm^−1^ correspond to the C–H stretching and bending vibration, respectively. The bands at 1324 cm^−1^ may be assigned to the C–N stretching vibration. The sharp absorption band at 1048 cm^−1^ is attributed to the stretching vibration of P–O–Zr, indicating the formation of zirconium phosphonate. The peaks at 728 and 579 cm^−1^ are attributed to the framework vibration of Zr–O–P and Zr–O bonds, respectively.^21^ The SEM image of Zr-ATMP is shown in Fig. 1B3, which shows aggregates of small particles.

The XRD pattern of Zr-PA hybrid (Fig. 1C1) shows some diffraction peaks at 5.8°, 11.6°, 22.9°, 33.9°, and 52.9°, indicating the crystalline nature of Zr-PA. However, these peaks are not as sharp as that of α-ZrP, probably due to the large PA group. The FTIR spectra of Zr-PA and PA are given in Fig. 1C2. In the FTIR spectra of PA, the peaks at 3384 cm^−1^ and 1642 cm^−1^ correspond to the stretching of OH from P–OH and water, respectively. The peaks at 2941 cm^−1^ and 2889 cm^−1^ are assigned to CH_2_ symmetric and asymmetric stretching of the PA ring. The peaks at 1197 cm^−1^, 982 cm^−1^, and 1043 cm^−1^ are assigned to the stretching of P=O, P–O, and (PO_3_)^2−^, respectively. In the FTIR spectra of Zr-PA hybrid, the typical bands of PA are observed at 1198 cm^−1^, 2938 cm^−1^, 2890 cm^−1^ corresponding to P=O, CH_2_ symmetric and asymmetric stretching, respectively. The band at 1046 cm^−1^ ((PO_3_)^2−^ stretching) is attributed to the stretching vibration of P–O–Zr, indicating the formation of zirconium phosphonate. The absorption bands at 526 and 714 cm^−1^ correspond to the framework vibration of Zr–O and Zr–O–P, respectively,^22,26^ which indicate that Zr-PA hybrid has been successfully prepared. The SEM image of Zr-PA is shown in Fig. 1C3, in which a number of particles of irregular shape are observed. The elemental composition analysis (ESI Table S1†) suggests that the mole ratios of Zr/ATMP and Zr/PA are 1 : 1 and 3 : 1 in Zr-ATMP and Zr-PA, respectively, and residual oxalic acid is also detected in the structure.

### 3.2 Flame retardant properties

The flame retardant properties of the composites were studied by LOI and CCT. The total content of the flame retardant additives was kept at 25 wt%, PE-*g*-MAH was used as a compatibilizer for the synergistic agents, and HDPE with PE-*g*-MAH was taken as the reference. The LOI values of the composites are presented in Table 1. The LOI value of the control sample (HD) without any flame retardant is found to be 18.1%. The addition of IFR composed of APP and THEIC (3 : 1 weight ratio) results in an increase in LOI value to 25.2%. The component of IFR is replaced by 3 wt% of zirconium-based compound, and the resulting HD/IFR/ZrP composite shows a LOI value of 24.8%, which is slightly lower than that of the composite containing only IFR. Compared to the HD/IFR/ZrP composite, the LOI value is reduced to 23.5% with the addition of Zr-PA, but increased to 26.2% with the addition of Zr-ATMP. Among the three synergistic agents tested in this study, Zr-ATMP shows the best flame retardant effect in terms of LOI. PA has been reported as a bio-based flame retardant for polylactic acid nonwoven fabric due to the high phosphorous content.^27^ PA is incorporated into the zirconium framework, and the resultant hybrid is expected to be useful flame retardant filler. However, the combination of Zr-PA with IFR results in a reduction in the flame-retardant performance of the composites compared to that containing only IFR.

**Fig. 2:**
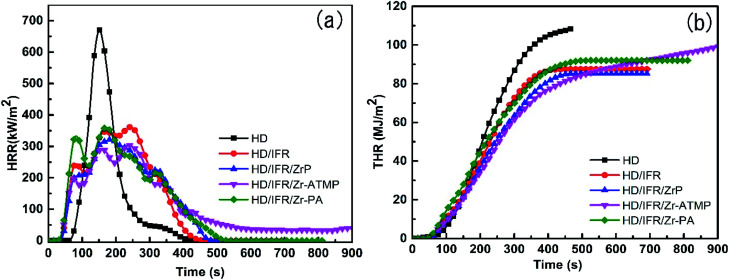
HRR (a) and THR (b) curves for IFR-HDPE composites.

The flame retardancy of the composites was also evaluated by CCT, which is considered a successful bench scale test to evaluate the flammability of materials and provides various parameters related to fire hazards such as heat release rate (HHR), total heat release rate (THR), peak heat release rate (PHRR), and mass loss rate (MRL). The PHRR and THR curves are presented in Fig. 2, and the data are collected in Table 2.

**Table 2:** Combustion performance obtained from CCT at a heat flux of 35 kW m^−2^

S. no.	Samples	PHRR (kW m^−2^)	THR (MJ m^−2^)	Av. sp. MLR (g s^−1^ m^−2^)	Av.-CO (kg kg^−1^)	Av.-CO_2_ (kg kg^−1^)	CO/CO_2_ ratio
1	HD	670.3	108.1	14.6	0.037	3.48	0.012
2	HD/IFR	361.6	87.5	8.7	0.086	3.46	0.025
3	HD/IFR/ZrP	321.6	85.1	8.1	0.072	3.59	0.020
4	HD/IFR/Zr-PA	359.1	92.0	8.2	0.091	4.25	0.021
5	HD/IFR/Zr-ATMP	304.1	99.4	7.5	0.092	3.69	0.025

HDPE burns rapidly and completely with nothing left at the end of the combustion, and the PHRR value is 670.3 kW m^−2^. The HRR curves of IFR composites with or without synergistic agent are very similar (Fig. 2a). The use of IFR based on APP and THEIC leads to a significant decrease in PHRR. During the combustion, a char layer is formed on the surface of the IFR composites which can act as an insulating barrier between fire and polymer matrix and thus prevent the exchange of heat and flammable gases. The PHRR of the HD/IFR composite containing only IFR is 361.6 kW m^−2^, which can be further decreased using synergistic agents. Among the three synergistic agents, Zr-ATMP results in a reduction of PHRR to 304.1 kW m^−2^, which is 54.6% and 16% lower than that of HD and HD/IFR composite, respectively. However, the use of Zr-PA as a synergistic agent leads to no significant reduction in PHRR compared to the HD/IFR composite, while the use of α-ZrP leads to 11% reduction in PHRR compared to the composite containing only IFR. The THR curves of the flame retardant composites are shown in Fig. 2(b). It is anticipated that the THR curves for flame retardant composites are lower than that of HD. However, the use of synergistic agents produces no significant reduction of THR at the end of the test. The final THR values of HD/IFR/Zr-PA and HD/IFR/Zr-ATMP composites are slightly higher than that of HD/IFR composite, but still lower than that of the control sample. Mathematically, THR is the area of the HRR curve, thus a longer and flatter HRR curve gives a higher THR value. The average specific mass loss rates (av. sp. MLR) of the flame retardant composites are lower than that of the control sample, and the minimum is observed in the composite containing Zr-ATMP. The CO and smoke production are strongly dependent on the materials and fire scenario. IFR produces an intumescent char on the surface of the polymer, which prevents the transfer of gas and thus leads to incomplete combustion and evolution of more CO. The average CO production of HD/IFR composite is higher than that of the control sample. Compared to the HD/IFR composite, the CO production is further increased with the use of zirconium-based compounds (Zr-ATMP and Zr-PA), but decreased with the use of α-ZrP. The intumescent char decomposes at high temperatures, and thus the average CO_2_ production is higher in the composites containing zirconium-based compounds. The CO/CO_2_ weight ratio of HD/IFR/ZrP and HD/IFR/Zr-PA composites is lower than that of HD/IFR composite, indicating that more CO_2_ is produced with the use of α-ZrP and Zr-PA. This is probably because the intumescent char is more likely to undergo surface oxidation at higher temperature. However, the use of Zr-ATMP produces a similar CO/CO_2_ weight ratio as that of the HD/IFR composite.

### 3.3 Thermal properties

**Fig. 3:**
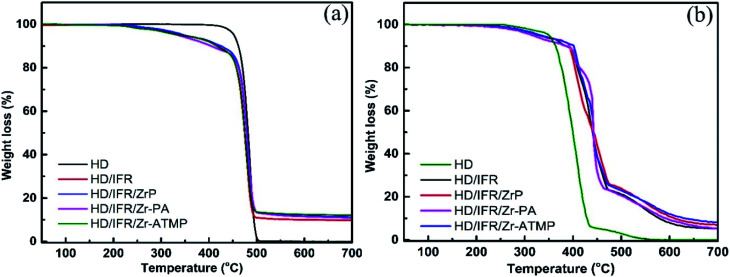
TGA curves for IFR-HDPE composites, in N_2_ (a) and in air (b) atmosphere.

The thermal degradation behaviors of the flame retardant composites were studied by TGA in a nitrogen atmosphere. The TGA curves are shown in Fig. 3a and the thermal degradation parameters are summarized in Table 3. In a non-oxidative environment, the main degradation mechanisms of HDPE involve chain scission and chain branching, which occur simultaneously and thus result in a single mass loss step.^28^ The initial decomposition temperature of 5% weight loss (*T*_5%_) of the flame retardant composites is lower than that of the control sample without flame retardant additives due to the early decomposition of the flame retardant additives. The addition of synergistic agents (Zr-PA and Zr-ATMP) leads to a further decrease in *T*_5%_, and the lowest *T*_5%_ is achieved in the composite containing Zr-PA. However, the composite containing α-ZrP shows a slight increase in *T*_5%_. The char residue of the flame retardant composites containing zirconium-based synergistic agent is increased, and those with Zr-ATMP show the highest char residue. The thermal degradation behaviors of the composites were also studied in an air atmosphere. The TGA curves are shown in Fig. 3(b) and the thermal degradation parameters are collected in Table 4. In an oxidative environment, the degradation process of HDPE primarily involves the radical chain reaction, and the TGA curve also shows some irregularities. PE begins to degrade earlier in the oxidative environment.^28^ The initial decomposition temperature (*T*_5%_) of the flame retardant composites is lower than that of the control sample. The addition of zirconium-based compounds in combination with IFR leads to only a marginal increase in *T*_5%_ and HD/IFR/Zr-PA composite shows the lowest *T*_5%_. The temperature of 50% weight loss (*T*_50%_) for HD/IFR is higher than that of the control sample, and the partial replacement of IFR with zirconium-based compounds leads to only a marginal increase in *T*_50%_. This suggests that the use of the IFR based on APP/THEIC can delay the degradation of the polymer and improve the thermal stability at high temperature in the oxidative environment.

**Table 3:** TGA analysis of the IFR-HDPE composites in N_2_ atmosphere

S. no.	Samples	*T*_5%_ (°C)	*T*_10%_ °C)	*T*_50%_ (°C)	Char (wt%)	
					600 °C	700 °C
1	HD	451.5	461.7	482.0	<0.1	<0.1
2	HD/IFR	344.1	418.7	474.8	10.1	9.7
3	HD/IFR-ZrP	349.1	424.2	479.7	11.7	11.3
4	HD/IFR/Zr-PA	334.1	406.2	479.5	11.6	10.9
5	HD/IFR/Zr-ATMP	339.1	416.1	475.5	12.5	12.1

**Table 4:** TGA analysis of the IFR-HDPE composites in air atmosphere

S. no.	Samples	*T*_5%_ (°C)	*T*_50%_ (°C)	Char (wt%)	
				600 °C	700 °C
1	HD	350.1	400.1	<0.1	<0.1
2	HD/IFR	329.6	440.1	8.1	5.4
3	HD/IFR/ZrP	331.5	442.1	10.9	7.0
4	HD/IFR/Zr-PA	314.6	442.1	8.0	5.4
5	HD/IFR/Zr-ATMP	333.6	442.3	12.1	8.1

**Fig. 4:**
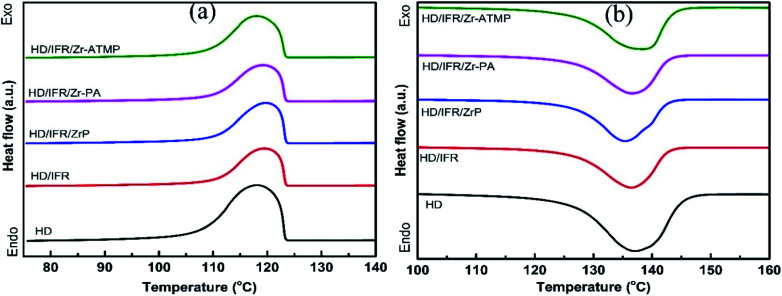
DSC curves of IFR-HDPE composites, cooling curves (a) and second heating curves (b).

The melting and crystallization behaviors of HDPE composites were determined by DSC, and the results are shown in Table 5. The melting and heating curves are presented in Fig. 4. The degree of crystallinity was calculated from the second melting enthalpy (Δ*H*_m_) using the following equation:
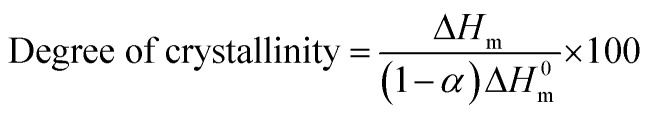
where Δ*H*_m_ is the melting enthalpy of samples (J g^−1^), Δ*H*^0^_m_ is the melting enthalpy of 100% crystalline form of HDPE (293 J g^−1^), and *α* is the mass fraction of flame retardants.^9^

**Table 5:** DSC results of IFR-HDPE composites

S. no.	Samples	*T*_m_ (°C)	*T*_mp_ (°C)	*T*_c_ (°C)	*T*_cp_ (°C)	Δ*H*_m_ (J g^−1^)	Δ*H*_c_ (J g^−1^)	Crystallization degree (%)
1	HD	126.5	137.2	123.3	118.1	211.3	224.3	72.1
2	HD/IFR	126.6	136.5	123.4	119.4	169.0	158.9	76.9
3	HD/IFR/ZrP	127.0	135.4	123.4	119.6	152.4	150.5	69.3
4	HD/IFR/Zr-PA	126.7	136.5	123.3	119.2	144.2	139.5	65.6
5	HD/IFR/Zr-ATMP	127.2	138.2	123.3	118.1	154.7	156.2	70.4

The crystallization onset temperature (*T*_c_) and peak temperature (*T*_cp_) of the flame retardant composites are close to that of the control sample, suggesting that the addition of flame retardants leads to no significant change in crystallization behaviors of the matrix. Notably, their melting onset temperature (*T*_m_) and peak temperature (*T*_mp_) are also close to that of the control sample. However, the use of IFR leads to an increase in the degree of crystallinity, probably due to the nucleating effect of APP for polyethylene spherulites. When different zirconium-based compounds are used as synergistic agents in combination with IFR, the degree of crystallinity is decreased and reaches a minimum in the composite containing Zr-PA. This is probably due to the inhibiting effect of the filler on crystallization, which in fact depends on the interfacial interaction between filler and matrix, filler content, particle size, *etc.*

### 3.4 Flame retardant mechanisms

#### 3.4.1 Morphology and chemical structure of the char residue

**Fig. 5:**
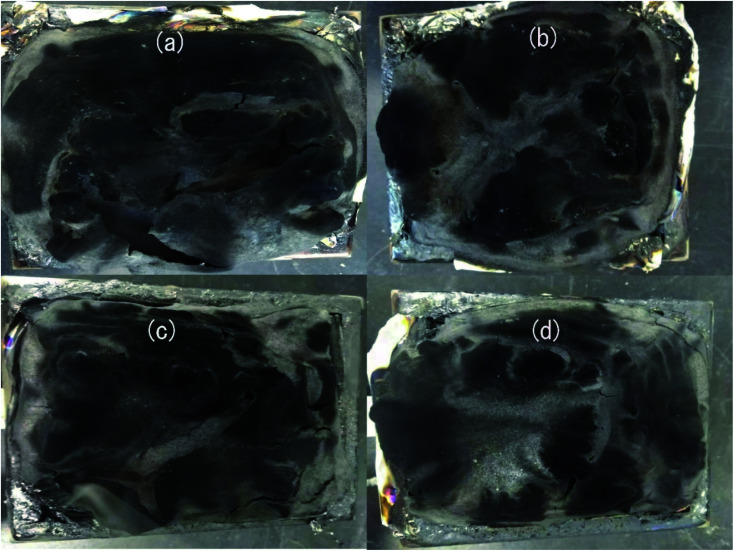
Digital images of the char residue of HD/IFR (a), HD/IFR/ZrP (b), HD/IFR/Zr-PA (c), and HD/IFR/Zr-ATMP (d) composites.

IFR acts in the condensed phase mechanism, and it can promote the formation of an intumescent char on the surface of the polymer that can prevent the transfer of heat and oxygen to the underlying polymer. Thus, the microstructure of the char plays a critical role in the flame retardant properties of the composites. The morphology and chemical compositions of the char residue were determined by SEM-EDX. The digital images of the char residue are shown in Fig. 5, and the compositions are shown in Table 6. Some cracks are observed on the surface of the char residue of HD/IFR and HD/IFR/Zr-PA composites (Fig. 5(a) and (c)), and a compact char surface is observed for the other two composites (Fig. 5(b) and (d)). The char residue of HD/IFR composite contains carbon, oxygen, nitrogen and phosphorous elements, but zirconium is also detected in the composites containing zirconium-based compounds. The increase in oxygen content in the char residue of the composites containing zirconium-based compounds is probably due to the formation of Zr–O linkage, while the decrease in carbon content is probably due to the surface oxidation of the char at higher temperatures.

**Table 6:** Compositions of the char residues from EDX analysis^a^

	HD/IFR		HD/IFR/ZrP		HD/IFR/Zr-PA		HD/IFR/Zr-ATMP	
	wt%	at%	wt%	at%	wt%	at%	wt%	at%
C	23.8	32.5	14.6	22.0	15.6	21.9	19.9	26.8
O	49.8	50.9	54.5	61.7	62.3	65.5	60.5	61.1
N	4.1	4.7	2.2	2.9	3.1	3.7	4.1	4.7
P	22.3	11.8	19.9	11.6	14.9	8.1	13.1	6.8
Zr	—	—	8.8	1.7	4.1	0.7	2.4	0.4

a Atomic percentage (at%), weight percentage (wt%).

**Fig. 6:**
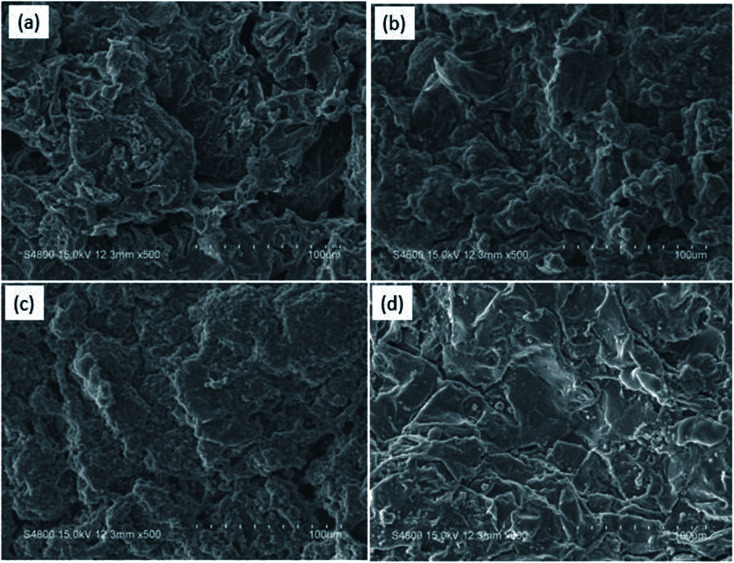
SEM images of the char residue after CCT test for HD/IFR (a), HD/IFR/ZrP (b) HD/IFR/Zr-PA (c), and HD/IFR/Zr-ATMP (d) composites.

The SEM images of the external surface of char residue after CCT test are shown in Fig. 6. Continuous, compact and foamy cell intumescent char is formed. Compared to HD/IFR/ZrP and HD/IFR/Zr-ATMP composites, the char surface of HD/IFR/Zr-PA composite is loose and porous, making it difficult to isolate the polymer from fire and thus leading to low flame retardant performance. However, the char residue of HD/IFR/Zr-ATMP composite is denser and more compact, which may lead to an increase in the flame retardant performance.

**Fig. 7:**
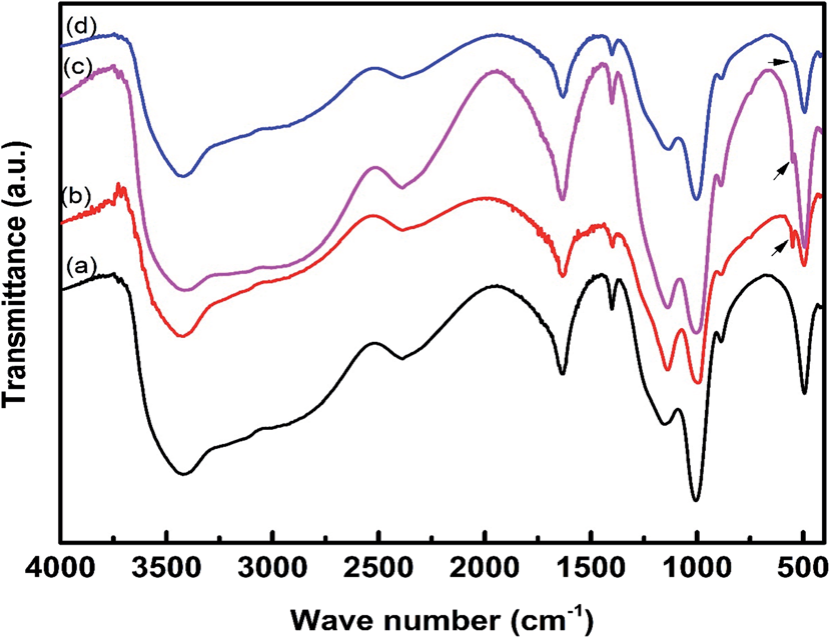
FTIR spectra of the char residue obtained after CCT test for HD/IFR (a), HD/IFR/ZrP (b), HD/IFR/Zr-ATMP (c), and HD/IFR/Zr-PA (d) composites.

The FTIR spectra of the char residue obtained from CCT test are presented in Fig. 7. The intumescent char formed by IFR consisting of APP and THEIC shows a poly-aromatic structure and phospho-carbonaceous structure linked *via* P–O–C bonds. The char residue also contains some phosphorous degradation products of different condensation degree as suggested by the presence of P–O–P structure.^9^ The FTIR spectra of the char residues of all composites are almost identical, indicating that these char residues have similar chemical structures. The band at 1150–1300 cm^−1^ corresponds to the P–O–C bond of the phospho–carbon complex, the band at around 1630 cm^−1^ is assigned to the aromatic structure, the band at 3420 cm^−1^ corresponds to the NH and/or OH structure, and the band at around 2390 cm^−1^ is assigned to –OH bond in the O=P–OH structure. The char residue of the composites containing zirconium-based compounds shows a small band at 550 cm^−1^ due to the Zr–O bond. It is important to note that this band is more prominent in HD/IFR/ZrP composite, but weak in HD/IFR/Zr-PA composite. The absorption band of Zr–O–P overlaps with that of P–O–C band.

#### 3.4.2 Raman analysis of char residue

**Fig. 8:**
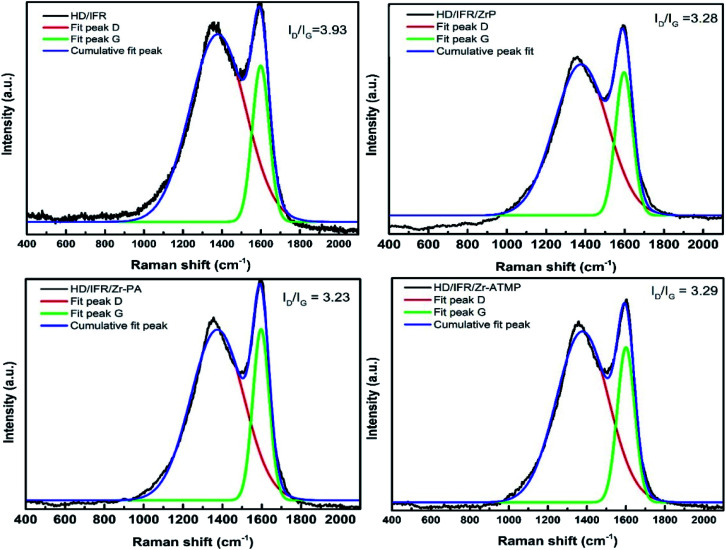
Raman spectra of the char residue of IFR-HDPE composites.

Raman spectroscopy was performed to characterize the graphitic structure of the char residue, where the G band represents E_2g_ mode of hexagonal graphite and the D band represents the defect and disorder in the graphitic layer. The intensity ratio of D to G band (*R* = *I*_D_/*I*_G_) is inversely proportional to the in-plane microcrystalline size and/or the in-plane phonon correlation length, and is considered a good estimate of the graphitization degree of carbon materials, such that the lower the *R* value is, the higher the graphitization degree of the char will be.^8,29^ The Raman spectra of the char residue of the flame retardant composites after CCT test are shown in Fig. 8. Each curve is subjected to peak fitting using Origin 9/Peak Fitting Module. The curves can be divided into two Gaussian bands, and the *R* values are evaluated. The results suggest that the use of zirconium-based compounds as synergistic agent leads to a decrease in the *R* values compared with the composite containing only IFR, suggesting the formation of char with a higher graphitic degree. It is also found that all zirconium-based compounds show almost similar *R* values, suggesting that the char residue has an identical graphitic structure. This means that zirconium-based compounds promote the formation of more graphitic char residue. The flame retardant performance of the composite containing Zr-PA is poorer than that of the composites containing α-ZrP and Zr-ATMP, but the graphitic degree of the char residue is almost the same. This suggests that the morphology of the char residue plays a crucial role, and the graphitic structure does not always act as an effective barrier to heat and oxygen. The morphology of the char is loose and porous for HD/IFR/Zr-PA composite, as shown in Fig. 6, which is a possible cause for its low flame retardant performance.

#### 3.4.3 TG-FTIR study

**Fig. 9:**
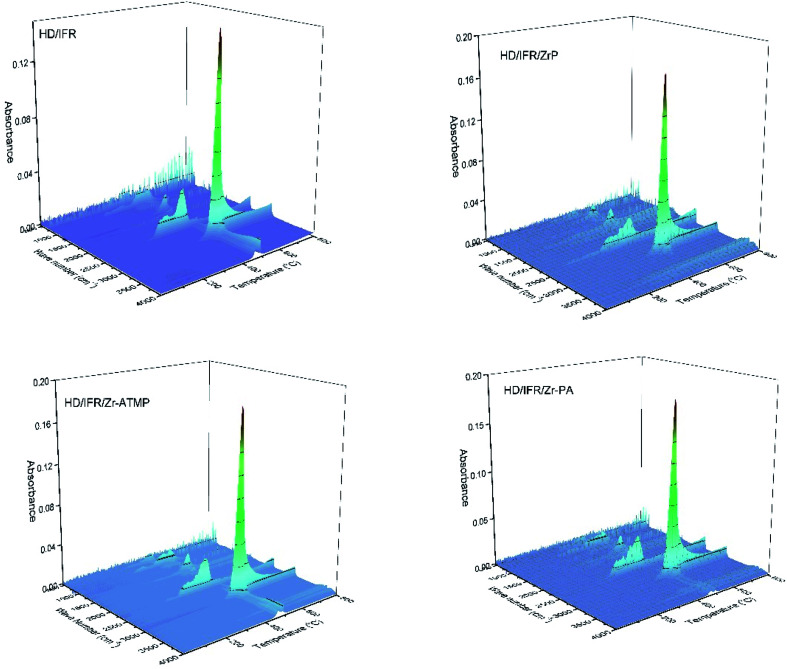
3D spectra of the pyrolysis volatile gaseous products of the IFR-HDPE composites.

**Fig. 10:**
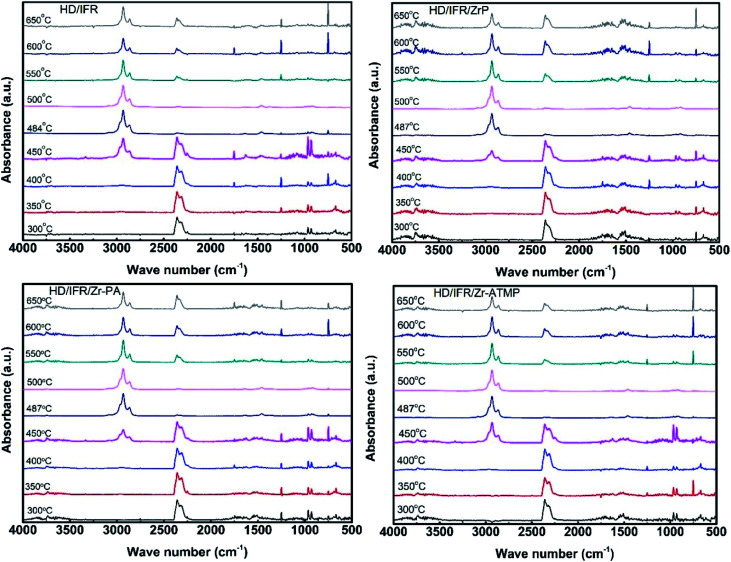
FTIR spectra of the volatile gaseous products of IFR-HDPE composites at different temperature.

To gain more insight into the effect of zirconium-based compounds on the thermal degradation behaviors of IFR-HDPE composites, the gaseous products were analyzed by TG-FTIR. Fig. 9 shows the 3D spectra and Fig. 10 shows the corresponding FTIR spectra at different temperatures. The FTIR spectra of the volatile gaseous products obtained from the composites containing different zirconium-based compounds show similar band positions, indicating the formation of same gaseous decomposition products. The strong absorption bands at 2930 and 2860 cm^−1^ correspond to C–H stretching, and the bands at 1352 and 1460 cm^−1^ correspond to the bending vibration of –CH_2_– and –CH_3_ groups, respectively.^30^ These bands are due to the decomposition of HDPE, which begins at about 400 °C in all composites. The TGA results suggest that pure HDPE is almost completely decomposed and only a negligible amount is left at over 500 °C. The absorption bands corresponding to the decomposition products of HDPE at higher temperatures suggest that the thermal degradation of the polymer is delayed by using flame retardants. Other major decomposition products include NH_3_ (960 and 930 cm^−1^), CO_2_ (2330 cm^−1^), water (3750 and 1600–1700 cm^−1^) and aliphatic (1258 and 746 cm^−1^).^31^ The production of NH_3_ at 300–450 °C is due to the decomposition of APP, while that of CO_2_ at 300–450 °C is due to the oxidation of the polymer and decomposition of THEIC. The production of CO_2_ at high temperature is probably due to the surface oxidation of the char residue. The similar FTIR spectra of volatile products suggest condensed phase flame retardant mechanism rather than gas phase even using zirconium-based compounds.

### 3.5 Mechanical properties

**Fig. 11:**
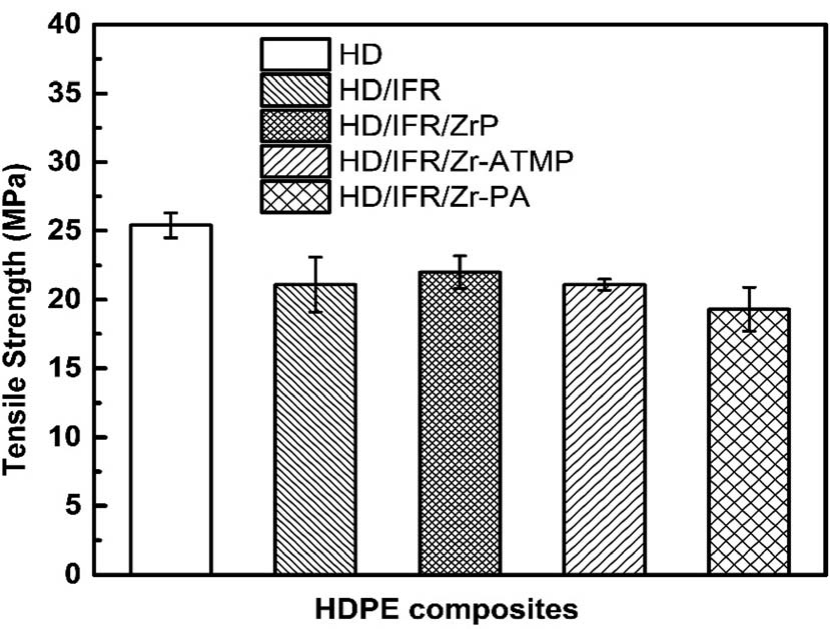
Tensile strength of IFR-HDPE composites.

The mechanical properties of the flame retardant composites are important for their applications. However, it remains a challenge to balance the mechanical and flame retardant properties. Fig. 11 indicates that the addition of IFR composed of APP and THEIC results in a decrease of the tensile strength of HDPE to 21.1 MPa, which is 17% lower than that of the control sample. The replacement of part of IFR with 3 wt% of zirconium-based compounds results in no significant change in the tensile strength compared to the composite containing only IFR. The tensile strength of HD/IFR/ZrP composite is slightly higher than that of HD/IFR composite, and the composite containing Zr-PA shows the lowest tensile strength of 19.3 MPa. IFR is usually hydrophilic and thus it can deteriorate the mechanical properties especially at a high loading. In the present study, PE-*g*-MAH is used as a compatibilizer and 80% of the tensile strength is retained. The use of zirconium-based compounds produces no significant improvement of the tensile strength of the composites.

## 4. Conclusions

In this study, zirconium-based compounds were used as flame retardant additives for IFR-HDPE composites. Zirconium phosphate and two organic–inorganic hybrids (zirconium organophosphonates) were synthesized using PA and ATMP and then combined with APP/THEIC to prepare IFR-HDPE composites. The HD/IFR/Zr-ATMP composite containing 3 wt% of Zr-ATMP shows a maximum LOI value of 26.2%. The combination of α-ZrP and Zr-ATMP leads to a further reduction of PHRR compared with the HD/IFR composite containing only IFR, whereas the use of Zr-PA produces no further reduction of PHRR. The use of Zr-ATMP in combination with IFR produces a compact char residue with more graphitic structure, which is responsible for the improvement of the flame retardant performance. However, the combination of zirconium-based compounds with IFR produces no significant improvement of the tensile strength compared to the composites containing only IFR.

## Conflicts of interest

There are no conflicts to declare.

## Supplementary Material

RA-010-D0RA04929H-s001
