# Supplementary material for: Effects of α-zirconium phosphate and zirconium organophosphonate on the thermal, mechanical and flame retardant properties of intumescent flame retardant high density polyethylene composites
Source: RSC Adv. 2020 Aug 21;10(51):30990–1002. doi: 10.1039/d0ra04929h (PMC9056347; doi:10.1039/d0ra04929h)
Supplement: RA-010-D0RA04929H-s001 [file RA-010-D0RA04929H-s001.pdf]

Electronic Supplementary Material (ESI) for RSC Advances.
This journal is © The Royal Society of Chemistry 2020

Supporting information

Effects of α-zirconium phosphate and zirconium organophosphonate on the thermal, mechanical and flame retardant properties of intumescent flame retardant high density polyethylene composites

*Santosh Khanal[1,3], Yunhua Lu[1], Li Dang[2], Muhammad Ali[1], *Shiai Xu[1, 2]*

[1]Shanghai Key Laboratory of Advanced Polymeric Materials, School of Materials Science and Engineering, East China University of Science and Technology, Shanghai 200237, China

[2]School of Chemical Engineering, Qinghai University, Xining 810016, China

[3]Central Department of Chemistry, Tribhuvan University, Kirtipur, Kathmandu, Nepal.

*Correspondence to: Shiai Xu (Tel: 86-021-64253353, E-mail: saxu@ecust.edu.cn)

1. Composition analysis of Zr-PA and Zr-ATMP

The compositional analysis of the Zr-PA and Zr-ATMP was performed by ICP-OES and conventional elemental analyzer and the results are shown in Table S1.

Table S1: Elemental analysis of the Zr-PA and Zr-ATMP

Samples	C (%)	H (%)	N (%)	P/Zr (mole ratio)	C/P (mole ratio)
Zr-ATMP	11.48	3.71	2.78	3.12	5.54
Zr-PA	15.78	4.51	-	1.86	3.31

The molar ratio of Zr/P is 1:3 and 1:2 in Zr-ATMP and Zr-PA respectively, suggesting that mole ratio of zirconium/ATMP and Zirconium/PA are in 1:1 and 3:1, respectively. A higher percentage of carbon suggests that residual oxalic acid is present in zirconium hybrid structure.

2. Thermogravimetric analysis of zirconium-based compounds

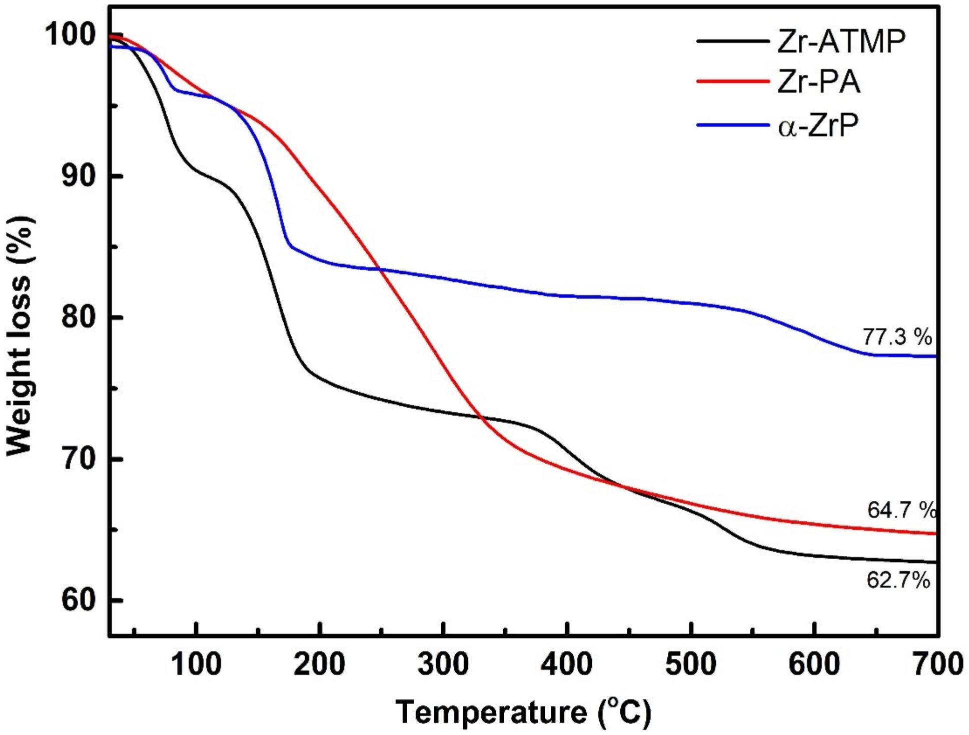

Figure S1: TGA curves for zirconium-based compounds in nitrogen atmosphere

The TGA curves for the zirconium based compounds in nitrogen are given in Fig S1.

The early weight loss in zirconium-based compounds is due to removal of surface adsorbed water. The residual oxalic acid present in Zr-PA and Zr-ATMP is also decomposed at lower temperature. The mass of residues are 77.3 %, 64.7 % and 62.7 % at 700 ^{o}C for α-ZrP, Zr-PA and Zr-ATMP respectively.
